# 1,8-Bis(4-fluoro­phen­yl)naphthalene

**DOI:** 10.1107/S1600536811020563

**Published:** 2011-06-11

**Authors:** Jin-Wu Feng, Qing-Chuan Han

**Affiliations:** a Department of Chemistry, Zhejiang University, Hangzhou 310027, People’s Republic of China

## Abstract

In the title compound, C_22_H_14_F_2_, the two benzene rings are oriented with respect to the naphthalene ring system at 67.76 (8) and 67.50 (8)°, and the two benzene rings are twisted with respect to each other at 18.95 (10)°. Weak inter­molecular C—H⋯π inter­actions are present in the crystal structure.

## Related literature

For related structures, see: Beagley *et al.* (1996 ▶); Wolf & Tumambac (2003 ▶).
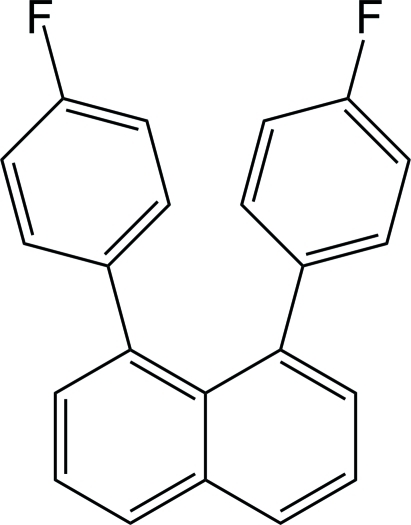


## Experimental

### 

#### Crystal data

C_22_H_14_F_2_
*M* *_r_* = 316.33
Triclinic, 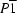

*a* = 8.4086 (11) Å
*b* = 9.6252 (11) Å
*c* = 11.2618 (13) Å
α = 87.705 (9)°
β = 74.895 (10)°
γ = 64.111 (12)°
*V* = 788.81 (16) Å^3^
*Z* = 2
Mo *K*α radiation
μ = 0.09 mm^−1^
*T* = 293 K
0.20 × 0.20 × 0.16 mm

#### Data collection

Oxford Diffraction Xcalibur Atlas Gemini ultra diffractometer
5315 measured reflections
2885 independent reflections
1543 reflections with *I* > 2σ(*I*)
*R*_int_ = 0.030

#### Refinement

*R*[*F* ^2^ > 2σ(*F* ^2^)] = 0.041
*wR*(*F* ^2^) = 0.095
*S* = 0.83
2885 reflections
217 parameters
H-atom parameters constrained
Δρ_max_ = 0.14 e Å^−3^
Δρ_min_ = −0.14 e Å^−3^

### 

Data collection: *CrysAlis PRO CCD* (Oxford Diffraction, 2009 ▶); cell refinement: *CrysAlis PRO CCD*; data reduction: *CrysAlis PRO RED* (Oxford Diffraction, 2009 ▶); program(s) used to solve structure: *SHELXTL* (Sheldrick, 2008 ▶); program(s) used to refine structure: *SHELXTL*; molecular graphics: *SHELXTL*; software used to prepare material for publication: *SHELXTL*.

## Supplementary Material

Crystal structure: contains datablock(s) I, global. DOI: 10.1107/S1600536811020563/xu5229sup1.cif

Structure factors: contains datablock(s) I. DOI: 10.1107/S1600536811020563/xu5229Isup2.hkl

Supplementary material file. DOI: 10.1107/S1600536811020563/xu5229Isup3.cdx

Supplementary material file. DOI: 10.1107/S1600536811020563/xu5229Isup4.cml

Additional supplementary materials:  crystallographic information; 3D view; checkCIF report

## Figures and Tables

**Table 1:** Hydrogen-bond geometry (Å, °) *Cg*1 and *Cg*2 are the centroids of the C7-benzene and C14-benzene rings.

*D*—H⋯*A*	*D*—H	H⋯*A*	*D*⋯*A*	*D*—H⋯*A*
C5—H5⋯*Cg*1^i^	0.93	2.88	3.6815 (19)	146
C22—H22⋯*Cg*2^ii^	0.93	2.84	3.6595 (19)	148

Symmetry codes: (i) 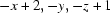; (ii) 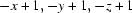.

**Fig. 1.**
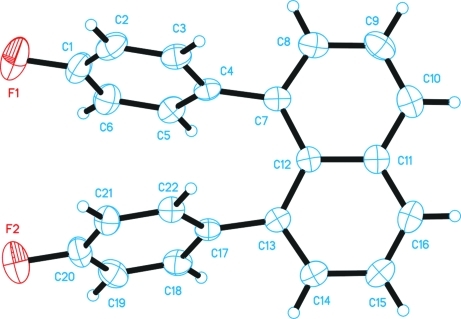
View of the molecule of (I) showing the atom-labelling scheme. Displacement ellipsoids are drawn at the 50% probability level.

**Fig. 2.**
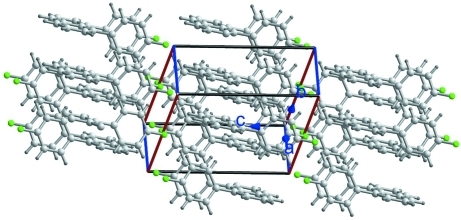
The crystal packing of the title compound viewed down the a axis.

## supplementary crystallographic information

### Comment

1,8-diaryl naphthalenes have attracted much attention mainly because of its U-shaped face-to-face geometry. These derivatives provide a special model to study the interactions of aromatic rings not only in the ground state but also in the transition state for rotation where they adopt an approximate edge-to-face conformation. Herein, we report 1,8-bis(4-fluorophenyl) naphthalene, a member of the 1,8-diaryl naphthalene family.

The asymmetric unit of the crystal structure contains one molecule featuring U-shaped geometry (Fig 1). The bond lengths and angles are normal and similar to those in related structures (Beagley *et al.*, 1996; Wolf & Tumambac, 2003). In the crystal structure, two phenyl rings twisting out of the naphthalene plane form dihedral angle of 18.95 (10)°. And the dihedral angles between the naphthalene and two phenyl rings are 67.76 (8)° and 67.50 (8)°.

### Experimental

1,8-Dibromonaphthalene (286 mg 1 mmol), 4-fluorophenylboronic acid (286 mg, 2.2 mmol), K_2_CO_3_ (1.38 g, 10 mmol) and Pd(PPh_3_)_4_ (5 mg, 0.004 mmol) in 50 ml of degassed toluene/ethanol (10:1) were mixed and refluxed for 12 h under N_2_, then filtrated. The filtrate was evaporated under reduced pressure. The crude products were purified by column chromatography (silica gel) from n-hexane as eluent to give the product in 92% yield (291 mg). Recrystallization from n-hexane/CH_2_Cl_2_ (5:1) gave colorless crystals.

### Refinement

H atoms were positioned geometrically with C—H = 0.93 Å, and constrained to ride on their parent atoms, with *U*_iso_(H) = 1.2*U*_eq_(C).

### Crystal data

**Table:**

C_22_H_14_F_2_	*Z* = 2
*M**_r_* = 316.33	*F*(000) = 328
Triclinic, *P*1	*D*_x_ = 1.332 Mg m^−^^3^
Hall symbol: -P 1	Mo *K*α radiation, λ = 0.71073 Å
*a* = 8.4086 (11) Å	Cell parameters from 1771 reflections
*b* = 9.6252 (11) Å	θ = 3.6–29.2°
*c* = 11.2618 (13) Å	µ = 0.09 mm^−^^1^
α = 87.705 (9)°	*T* = 293 K
β = 74.895 (10)°	Block, colorless
γ = 64.111 (12)°	0.20 × 0.20 × 0.16 mm
*V* = 788.81 (16) Å^3^	

### Data collection

**Table:**

Oxford Diffraction Xcalibur Atlas Gemini ultra diffractometer	1543 reflections with *I* > 2σ(*I*)
Radiation source: fine-focus sealed tube	*R*_int_ = 0.030
graphite	θ_max_ = 25.3°, θ_min_ = 3.6°
Detector resolution: 10.3592 pixels mm^-1^	*h* = −10→7
ω scans	*k* = −11→11
5315 measured reflections	*l* = −13→13
2885 independent reflections	

### Refinement

**Table:**

Refinement on *F*^2^	Primary atom site location: structure-invariant direct methods
Least-squares matrix: full	Secondary atom site location: difference Fourier map
*R*[*F*^2^ > 2σ(*F*^2^)] = 0.041	Hydrogen site location: inferred from neighbouring sites
*wR*(*F*^2^) = 0.095	H-atom parameters constrained
*S* = 0.83	*w* = 1/[σ^2^(*F*_o_^2^) + (0.0473*P*)^2^] where *P* = (*F*_o_^2^ + 2*F*_c_^2^)/3
2885 reflections	(Δ/σ)_max_ < 0.001
217 parameters	Δρ_max_ = 0.14 e Å^−^^3^
0 restraints	Δρ_min_ = −0.14 e Å^−^^3^

### Special details

**Table:**

Geometry. All e.s.d.'s (except the e.s.d. in the dihedral angle between two l.s. planes) are estimated using the full covariance matrix. The cell e.s.d.'s are taken into account individually in the estimation of e.s.d.'s in distances, angles and torsion angles; correlations between e.s.d.'s in cell parameters are only used when they are defined by crystal symmetry. An approximate (isotropic) treatment of cell e.s.d.'s is used for estimating e.s.d.'s involving l.s. planes.
Refinement. Refinement of *F*^2^ against ALL reflections. The weighted *R*-factor *wR* and goodness of fit *S* are based on *F*^2^, conventional *R*-factors *R* are based on *F*, with *F* set to zero for negative *F*^2^. The threshold expression of *F*^2^ > σ(*F*^2^) is used only for calculating *R*-factors(gt) *etc*. and is not relevant to the choice of reflections for refinement. *R*-factors based on *F*^2^ are statistically about twice as large as those based on *F*, and *R*- factors based on ALL data will be even larger.

### Fractional atomic coordinates and isotropic or equivalent isotropic displacement parameters (Å^2^)

**Table:**

	*x*	*y*	*z*	*U*_iso_*/*U*_eq_	
F1	0.37936 (19)	0.21582 (15)	0.99923 (10)	0.1062 (5)	
F2	0.78304 (18)	0.38981 (15)	0.97949 (10)	0.0976 (4)	
C1	0.4395 (3)	0.2123 (3)	0.87403 (17)	0.0661 (6)	
C2	0.3265 (3)	0.3170 (2)	0.81271 (19)	0.0677 (6)	
H2	0.2094	0.3895	0.8549	0.081*	
C3	0.3897 (2)	0.3131 (2)	0.68655 (17)	0.0572 (5)	
H3	0.3129	0.3828	0.6432	0.069*	
C4	0.5657 (2)	0.20736 (19)	0.62257 (15)	0.0446 (4)	
C5	0.6740 (2)	0.10154 (19)	0.69004 (15)	0.0499 (5)	
H5	0.7910	0.0276	0.6492	0.060*	
C6	0.6115 (3)	0.1040 (2)	0.81600 (17)	0.0608 (5)	
H6	0.6852	0.0332	0.8605	0.073*	
C7	0.6285 (2)	0.20390 (18)	0.48640 (15)	0.0437 (4)	
C8	0.5392 (2)	0.16090 (19)	0.42018 (17)	0.0552 (5)	
H8	0.4422	0.1405	0.4634	0.066*	
C9	0.5858 (3)	0.1461 (2)	0.29184 (18)	0.0635 (5)	
H9	0.5219	0.1156	0.2509	0.076*	
C10	0.7243 (3)	0.1765 (2)	0.22772 (17)	0.0604 (5)	
H10	0.7570	0.1653	0.1419	0.072*	
C11	0.8212 (2)	0.22514 (18)	0.28818 (15)	0.0487 (5)	
C12	0.7750 (2)	0.24087 (17)	0.41961 (14)	0.0409 (4)	
C13	0.8780 (2)	0.29298 (18)	0.47501 (14)	0.0423 (4)	
C14	1.0158 (2)	0.32195 (19)	0.39941 (15)	0.0526 (5)	
H14	1.0830	0.3540	0.4358	0.063*	
C15	1.0593 (3)	0.3056 (2)	0.27130 (17)	0.0588 (5)	
H15	1.1533	0.3267	0.2236	0.071*	
C16	0.9638 (3)	0.2587 (2)	0.21706 (16)	0.0582 (5)	
H16	0.9921	0.2482	0.1314	0.070*	
C17	0.8487 (2)	0.31863 (18)	0.60942 (14)	0.0427 (4)	
C18	0.9840 (2)	0.2255 (2)	0.66502 (16)	0.0546 (5)	
H18	1.0912	0.1458	0.6175	0.066*	
C19	0.9624 (3)	0.2488 (2)	0.78916 (18)	0.0626 (5)	
H19	1.0528	0.1852	0.8262	0.075*	
C20	0.8053 (3)	0.3675 (2)	0.85619 (16)	0.0600 (5)	
C21	0.6700 (3)	0.4637 (2)	0.80646 (16)	0.0553 (5)	
H21	0.5646	0.5439	0.8551	0.066*	
C22	0.6922 (2)	0.44001 (18)	0.68275 (15)	0.0465 (4)	
H22	0.6013	0.5060	0.6471	0.056*	

### Atomic displacement parameters (Å^2^)

**Table:**

	*U*^11^	*U*^22^	*U*^33^	*U*^12^	*U*^13^	*U*^23^
F1	0.1273 (12)	0.1060 (11)	0.0602 (8)	−0.0503 (9)	0.0131 (7)	0.0029 (7)
F2	0.1270 (12)	0.1024 (10)	0.0591 (8)	−0.0398 (9)	−0.0378 (7)	0.0068 (7)
C1	0.0790 (16)	0.0652 (15)	0.0519 (12)	−0.0405 (14)	0.0016 (12)	0.0036 (11)
C2	0.0498 (13)	0.0642 (14)	0.0749 (15)	−0.0241 (12)	0.0053 (11)	−0.0058 (11)
C3	0.0432 (12)	0.0519 (12)	0.0719 (13)	−0.0182 (10)	−0.0131 (10)	0.0039 (9)
C4	0.0410 (11)	0.0397 (10)	0.0558 (11)	−0.0217 (9)	−0.0111 (9)	0.0068 (9)
C5	0.0503 (11)	0.0371 (10)	0.0570 (12)	−0.0177 (9)	−0.0094 (9)	0.0070 (9)
C6	0.0715 (15)	0.0505 (12)	0.0578 (12)	−0.0269 (12)	−0.0145 (11)	0.0139 (10)
C7	0.0404 (10)	0.0303 (9)	0.0581 (11)	−0.0118 (8)	−0.0174 (8)	0.0092 (8)
C8	0.0550 (12)	0.0449 (11)	0.0720 (13)	−0.0234 (10)	−0.0261 (10)	0.0143 (9)
C9	0.0759 (15)	0.0502 (12)	0.0771 (14)	−0.0291 (12)	−0.0390 (12)	0.0076 (10)
C10	0.0740 (14)	0.0464 (12)	0.0569 (12)	−0.0189 (11)	−0.0253 (11)	0.0049 (9)
C11	0.0523 (12)	0.0334 (10)	0.0520 (11)	−0.0107 (9)	−0.0160 (9)	0.0064 (8)
C12	0.0399 (10)	0.0284 (9)	0.0472 (10)	−0.0073 (8)	−0.0143 (8)	0.0054 (7)
C13	0.0374 (10)	0.0312 (9)	0.0508 (10)	−0.0096 (8)	−0.0098 (8)	0.0045 (8)
C14	0.0479 (11)	0.0458 (11)	0.0600 (12)	−0.0201 (9)	−0.0092 (9)	0.0044 (9)
C15	0.0517 (12)	0.0512 (12)	0.0629 (13)	−0.0212 (10)	−0.0020 (10)	0.0090 (9)
C16	0.0573 (13)	0.0484 (12)	0.0509 (11)	−0.0125 (10)	−0.0058 (10)	0.0073 (9)
C17	0.0418 (11)	0.0370 (10)	0.0528 (11)	−0.0202 (9)	−0.0139 (9)	0.0058 (8)
C18	0.0427 (11)	0.0488 (12)	0.0661 (13)	−0.0144 (9)	−0.0145 (9)	0.0015 (9)
C19	0.0625 (14)	0.0600 (13)	0.0706 (14)	−0.0233 (12)	−0.0340 (11)	0.0129 (11)
C20	0.0782 (16)	0.0618 (14)	0.0466 (12)	−0.0331 (13)	−0.0236 (11)	0.0076 (10)
C21	0.0617 (13)	0.0444 (11)	0.0527 (12)	−0.0189 (10)	−0.0114 (10)	−0.0011 (9)
C22	0.0473 (11)	0.0341 (10)	0.0550 (11)	−0.0145 (9)	−0.0154 (9)	0.0060 (8)

### Geometric parameters (Å, °)

**Table:**

F1—C1	1.3646 (19)	C11—C16	1.414 (2)
F2—C20	1.3648 (19)	C11—C12	1.425 (2)
C1—C2	1.358 (3)	C12—C13	1.441 (2)
C1—C6	1.361 (3)	C13—C14	1.376 (2)
C2—C3	1.377 (2)	C13—C17	1.482 (2)
C2—H2	0.9300	C14—C15	1.390 (2)
C3—C4	1.390 (2)	C14—H14	0.9300
C3—H3	0.9300	C15—C16	1.348 (2)
C4—C5	1.390 (2)	C15—H15	0.9300
C4—C7	1.483 (2)	C16—H16	0.9300
C5—C6	1.375 (2)	C17—C18	1.388 (2)
C5—H5	0.9300	C17—C22	1.395 (2)
C6—H6	0.9300	C18—C19	1.377 (2)
C7—C8	1.371 (2)	C18—H18	0.9300
C7—C12	1.445 (2)	C19—C20	1.361 (3)
C8—C9	1.391 (2)	C19—H19	0.9300
C8—H8	0.9300	C20—C21	1.355 (3)
C9—C10	1.344 (2)	C21—C22	1.371 (2)
C9—H9	0.9300	C21—H21	0.9300
C10—C11	1.412 (2)	C22—H22	0.9300
C10—H10	0.9300		
			
C2—C1—C6	122.77 (18)	C11—C12—C13	117.21 (14)
C2—C1—F1	119.0 (2)	C11—C12—C7	117.47 (15)
C6—C1—F1	118.2 (2)	C13—C12—C7	125.33 (14)
C1—C2—C3	118.37 (19)	C14—C13—C12	118.86 (15)
C1—C2—H2	120.8	C14—C13—C17	115.85 (15)
C3—C2—H2	120.8	C12—C13—C17	125.28 (14)
C2—C3—C4	121.43 (18)	C13—C14—C15	123.13 (17)
C2—C3—H3	119.3	C13—C14—H14	118.4
C4—C3—H3	119.3	C15—C14—H14	118.4
C5—C4—C3	117.57 (16)	C16—C15—C14	119.22 (17)
C5—C4—C7	122.12 (15)	C16—C15—H15	120.4
C3—C4—C7	120.21 (16)	C14—C15—H15	120.4
C6—C5—C4	121.32 (17)	C15—C16—C11	121.14 (17)
C6—C5—H5	119.3	C15—C16—H16	119.4
C4—C5—H5	119.3	C11—C16—H16	119.4
C1—C6—C5	118.50 (18)	C18—C17—C22	117.78 (15)
C1—C6—H6	120.8	C18—C17—C13	119.94 (15)
C5—C6—H6	120.8	C22—C17—C13	122.19 (15)
C8—C7—C12	118.37 (15)	C19—C18—C17	121.23 (17)
C8—C7—C4	116.25 (15)	C19—C18—H18	119.4
C12—C7—C4	125.38 (14)	C17—C18—H18	119.4
C7—C8—C9	123.50 (17)	C20—C19—C18	118.20 (18)
C7—C8—H8	118.2	C20—C19—H19	120.9
C9—C8—H8	118.2	C18—C19—H19	120.9
C10—C9—C8	119.15 (18)	C21—C20—C19	123.14 (17)
C10—C9—H9	120.4	C21—C20—F2	118.63 (18)
C8—C9—H9	120.4	C19—C20—F2	118.23 (18)
C9—C10—C11	121.25 (17)	C20—C21—C22	118.43 (17)
C9—C10—H10	119.4	C20—C21—H21	120.8
C11—C10—H10	119.4	C22—C21—H21	120.8
C10—C11—C16	119.35 (17)	C21—C22—C17	121.20 (16)
C10—C11—C12	120.23 (16)	C21—C22—H22	119.4
C16—C11—C12	120.43 (16)	C17—C22—H22	119.4

### Hydrogen-bond geometry (Å, °)

**Table:**

Cg1 and Cg2 are the centroids of the C7-benzene and C14-benzene rings.

**Table:**

*D*—H···*A*	*D*—H	H···*A*	*D*···*A*	*D*—H···*A*
C5—H5···Cg1^i^	0.93	2.88	3.6815 (19)	146
C22—H22···Cg2^ii^	0.93	2.84	3.6595 (19)	148

Symmetry codes: (i) −*x*+2, −*y*, −*z*+1; (ii) −*x*+1, −*y*+1, −*z*+1.
